# Eye Symptoms of Infantile Scurvy

**Published:** 1905-09

**Authors:** Irving Snow

**Affiliations:** Buffalo, N. Y.


### Eye Symptoms of Infantile Scurvy.

### A Case of Infantile Scurvy With Extreme Protrusion of the Right Eyeball, Shown by Autopsy to be Due to a Large Retrobulbar Hematoma.[1]

By IRVING SNOW, M. D., Buffalo, N. Y.

THE eye symptoms of infantile scurvy receive small notice in works on ophthalmology, and the writer has recently seen a consultation of eight oculists result in much difference of opinion as to the significance, cause, and treatment of proptosis in a scorbutic baby.

The history of the case is interesting, as it is one of the few where the cause of the protrusion of the eyeball was shown by autopsy to be an extensive subperiosteal hemorrhage of the orbital bones.

The patient was the offspring of healthy young parents, who had two older healthy children. It was born after a normal labor. It showed an intolerance of cow's milk, and was given cereal milk, upon which it throve for nine months, when it developed gradually pain, swelling and pseudoparalysis in both lower extremities. The loss of power was attributed to an obscure spinal injury by the family physician. Some weeks later, a slight protrusion of the left eyeball appeared, associated with blackened lids. Ten days later, a sudden protrusion of the right eyeball occurred, so extreme in degree that the closed lids left a large uncovered space on the cornea; both lids had a dark, bruised-like color, and were much swollen. Four days afterwards the baby became feverish and loked very ill.

The child was seen at 9 p. m., January 18, by Dr. Lorenzo Burrows, who decided that the extreme proptosis of the right eye was not due to sarcoma, orbital cellulitis, or fracture of the skull, and referred the matter to Dr. Irving Snow, as a medical case.

---

1. Read by title at the seventeenth annual meeting of the American Pediatric Society, Lake George, N. Y., June 20, 1905. [*Archives of Pediatrics*, August, 1905.]

*January 19.*—Examination by Dr. Irving Snow, 9 a. m. Child very apathetic; good development; slight rachitic rosary, heart rapid and weak; nothing in chest or abdomen; temperature, 100° F.

Right eyeball protruding as in the most extreme case of exophthalmic goitre; cornea cloudy; upper and lower eyelids black and swollen, and do not cover the eyeball; no chemosis; eyeball freely movable; no discoloration of conjunctiva.

Moderate proptosis of left eyeball, with swollen, discolored lids; left cornea clear.

Gums slightly swollen and violet colored. No teeth.

Upper extremities, normal.

Legs below knee uniformly swollen and painful. Feet edematous. No purpuric spots or heat of surface.

The child thus presented a typical picture of infantile scurvy. The proptosis and swelling and pseudoparalysis of the legs were due to subperiosteal hemorrhage. Orange-juice, fresh milk and stimulants were given, but the temperature rose to 107.5° F. at 3.30 p. m. The child showed increasing prostration, and died at 11 p. m., after a stay of twenty-six hours in the hospital.

*Autopsy.*—An examination of only right eye permitted. A huge hematoma lay between the periosteum and bone of the orbit, filling the pyramidal space behind the eye and extending nearly around the entire orbit, stripping off nearly the whole orbital periosteum. No retinal hemorrhages. A smear from the clot showed large quantities of pus containing a bacillus like the influenza bacillus. Subperiosteal hemorrhage occurred in both orbits—in the left orbit, earlier and slighter in degree, and spontaneously ceasing. In the right orbit, an extensive, sudden hemorrhage took place, pushing forward the eyeball.

The rise of temperature in the last few hours of life was due to some terminal infection, probably proceeding from the infected retrobulbar hematoma.

The question of operative relief of the exophthalmos by removing the clot behind the eyeball was discussed, but was dismissed as unpractical on account of the moribund condition of the patient and the usual spontaneous absorption of the clot in a more favorable case.

Of the collective investigations of the American Pediatric Society[1] of 340 cases of scurvy, eye symptoms were mentioned in 49; a swelling mentioned in 9; in 18 protrusion of the eyeball; and in 22 both symptoms occurred. In the six autopsies reported by the collective investigation, in none was an examination of the eye made. Heubner[2] reports 65 cases of infantile scurvy, orbit involved four times.

Regarding adult scurvy, Buzzard[3] (Reynolds's System) says, in some cases at an early period: "The integument around one or both orbits is puffed up into a bruise-colored swelling; the conjunctiva covering the sclerotic is turned and of a brilliant red color throughout; the cornea lies at the bottom of a circular trench or well. There is nothing inflammatory about the condition. It resembles a very violent ophthalmia in color, but with no pain or discharge."

Sudden exophthalmos in a baby should at once excite suspicions of scurvy. It may, in fact, be the first indication of the disease. The rapidity with which the proptosis occurs indicates a hemorrhagic lesion.

Barlow[4] (Grancher's System) says of infantile scurvy: "A remarkable symptom in one or both orbits is not rare. Suddenly, without premonitory sign or appreciable cause, we find a moderate exophthalmos, with the ocular globes turned downward. This symptom appears and augments for twenty-four hours. When it has reached its acme, one finds a deep ecchymosis and thickening of the superior lids, due to a hemorrhagic effusion. Both orbits may be affected, but successively and in an unequal manner. The proptosis may be only slight. There is no increase of intraocular tension or hemorrhage into the deeper parts of the eye. The eyeball is freely movable, not anchored by an inflammatory exudation, as in orbital cellulitis. The hemorrhage effusion into the eyelids affects the deeper parts; the conjunctiva of the lid and globe are only occasionally affected.

Similar cases of scorbutic proptosis have been collected, as follows:

Barlow[5] (Keating's System) describes 2 cases of scorbutic proptosis. He says it is dependent on a bone lesion, an extravasation of blood between the orbital plate of the frontal and its subjacent periosteum, the extravasation pushing out the eyeball.

(1) Eight-months-old baby, brought on account of proptosis of one eyeball. Great tenderness in the limbs, and general cachexia. Since early infancy the baby has been rather tender in the legs. During the last week the tenderness became excessive; both upper eyelids became suddenly swollen three weeks ago, the right upper lid purplish-red color, due to deep extravasation into its substance. No ecchymosis of palpebral of ocular conjunctiva. Slight proptosis of right eye. Cure after antiscorbutics.

(2) Ten-months-old baby, artifically fed. Legs tender at eighth month; at nine months the wrists became tender; there later developed ecchymosis in both eyelids; pseudoparalysis and slight proptosis of life eye.

Child died after an illness of three months. The autopsy showed subperiosteal hemorrhages. Eye not examined.

(3) Nicholai.[6] Nederlausch. Tijdschrift Vor Geneekunde. Child nine months, brought to Nicholai for a sudden protrusion of the left eye. On both upper eyelids, there was a small greenish-yellow spot. Child otherwise well. Two days after Nicholai's first examination, a neuroparalytic keratitis developed, and Nicholai found in the gums the characteristic changes of scurvy. A few days later a retrobulbar hemorrhage occurred in the right eye. On antiseptic washes and antiscorbutics the keratitis and ocular protrusion disappeared.

(4) Ed. Meyer.[7] Seven-months-old child, previously well, attacked with edematous swelling and pain in left ankle; left-sided exophthalmos. Death with increasing anemia. Autopsy.—Rachitis, anemia, hemorrhagic pachymeningitis, hemorrhagic periostitis of the left orbit and tibia, fatty myocarditis, hemorrhages into heart, lungs and kidney.

(5) Ashby and Wright.[8] Girl, seven months old, fed on dried milk and maltose-food for seven months. Throve and looked like a prize baby. Bad cough for two to three weeks. Two weeks before examination, the left eyeball became suddenly prominent, and protruded so much that the nurse thought it would drop out. The proptosis was attributed to a slight blow on the eye the baby received from a comforter. The eye had been prominent for two weeks, during which time the baby had several bad fainting attacks. The baby was large and fat and pale; temperature 101° F.; drowsy, but readily aroused. The eyeball was exposed in part; the lids when closed did not meet; lids not puffy or ecchymosed; ribs beaded; no teeth, gums normal. Urine stained the diaper brown. Rapid improvement on fresh milk and orange-juice.

It is interesting to note that so acute and experienced an observer as Dr. Nansen[9] looks on adult scurvy as a disease not due to the absence of a certain element in the food, but to the presence of ptomaines in badly preserved salt beef or other preserved foods. He believes that with sterilised foods and properly given, there would be no scurvy, and that orange and lime juice are unnecessary. Both Dr. Nansen and Dr. Caillé believe that scurvy in adults and children is due to ptomaine poisoning.

### BIBLIOGRAPHY.

1. Transactions American Pediatric Society, 1898.
2. Heubner.
3. Reynolds' System, Vol, I., p. 445.
4. Grancher's System, Vol. II., p. 175.
5. Keating's System, Vol, II., p. 275.
6. Nederlausch. Tijsdchrift Vor Geneekunde.
7. Berliner klinische Wochenschrift, 1896, p. 4.
8. Ashby and Wright, p. 195.
9. Ashby and Wright, p. 193.

